# An effective docking strategy for virtual screening based on multi-objective optimization algorithm

**DOI:** 10.1186/1471-2105-10-58

**Published:** 2009-02-11

**Authors:** Honglin Li, Hailei Zhang, Mingyue Zheng, Jie Luo, Ling Kang, Xiaofeng Liu, Xicheng Wang, Hualiang Jiang

**Affiliations:** 1 School of Pharmacy, East China University of Science and Technology, Shanghai 200237, PR China; 2 Drug Discovery and Design Center, State Key Laboratory of Drug Research, Shanghai Institute of Materia Medica, Chinese Academy of Sciences, Shanghai 201203, PR China; 3 Department of Engineering Mechanics, State Key Laboratory of Structural Analyses for Industrial Equipment, Dalian University of Technology, Dalian 116023, PR China; 4 Department of Medical Oncology, Dana-Farber Cancer Institute and Harvard Medical School, Boston, MA 02115, USA; 5 School of Information Science and Engineering, East China University of Science and Technology, Shanghai 200237, PR China

## Abstract

**Background:**

Development of a fast and accurate scoring function in virtual screening remains a hot issue in current computer-aided drug research. Different scoring functions focus on diverse aspects of ligand binding, and no single scoring can satisfy the peculiarities of each target system. Therefore, the idea of a consensus score strategy was put forward. Integrating several scoring functions, consensus score re-assesses the docked conformations using a primary scoring function. However, it is not really robust and efficient from the perspective of optimization. Furthermore, to date, the majority of available methods are still based on single objective optimization design.

**Results:**

In this paper, two multi-objective optimization methods, called MOSFOM, were developed for virtual screening, which simultaneously consider both the energy score and the contact score. Results suggest that MOSFOM can effectively enhance enrichment and performance compared with a single score. For three different kinds of binding sites, MOSFOM displays an excellent ability to differentiate active compounds through energy and shape complementarity. EFMOGA performed particularly well in the top 2% of database for all three cases, whereas MOEA_Nrg and MOEA_Cnt performed better than the corresponding individual scoring functions if the appropriate type of binding site was selected.

**Conclusion:**

The multi-objective optimization method was successfully applied in virtual screening with two different scoring functions that can yield reasonable binding poses and can furthermore, be ranked with the potentially compromised conformations of each compound, abandoning those conformations that can not satisfy overall objective functions.

## Background

With the thriving development and confirmative significance of computational chemistry in drug discovery, more and more medicinal chemists and pharmacologists are using computational methods in their drug discovery research[1,2], and numerous drugs developed based on the clues provided by computations (modeling and simulation) have entered clinical trials or have been launched into the market already[3]. For the computational chemist, an attractive goal is to develop computer programs capable of automatically evaluating large-scale chemical libraries (databases). These computational methods are referred to as virtual screening (VS)[4]. In general, two strategies have been employed in virtual screening: (1), using pharmacophore-based database searching (PBDS) methods to identify potential hits from chemical libraries, mostly in the cases where three-dimensional (3D) structures of the targets are unknown; and (2), using molecular docking approaches to screen the libraries in cases where the 3D structures of the targets are available[4,5]. Because more and more 3D structures of drug target proteins are available, VS with molecular docking approaches has become promising, as demonstrated by numerous recent examples[2,6-10].

The core steps of structure-based virtual screening (SBVS) are docking and scoring. Since Kuntz *et al*.[11] published the first docking algorithm DOCK in 1982, numerous docking programs have been developed during the past two decades [12-25]. Several comprehensive reviews of the advances of docking algorithms and applications have been published [26-30]. Scoring (ranking) the compounds retrieved from a database is performed simultaneously with the docking simulation. Molecular docking is a typical optimization problem, for it is difficult to obtain the global optimum solution. As the fitness during the optimization process, scoring function should be fast and accurate enough, allowing simultaneous ranking of the retrieved poses in the optimization process. Based on this idea, several scoring functions have been developed [31-33]. Unfortunately, there is no scoring function developed so far that can reliably and consistently predict a ligand-protein binding mode and the binding affinity at the same time[31,32,34]. Therefore, heuristic docking and consensus score strategies are often used in virtual screening [34-36].

Since a huge number of compounds in a database have to be thoroughly tested in the virtual screening process, several crucial issues have to be addressed. One is the computational cost; only docking programs capable of docking a flexible ligand within a reasonable time scale are acceptable for virtual screening[34]. The other one is the ability to discriminate between true actives and inactive compounds; only those docking approaches able to distinguish the active molecules rapidly and accurately, are suitable for virtual screening applications in practice. Although the consensus score strategy has demonstrated an advantage over single scores, it is actually based on the results of single scoring optimization. This means that consensus scoring only re-scores a limited molecules or conformations (generally 30 or more), thereby inevitably losing a number of true positives[33,34]. To some extent, consensus scoring seems to be far-fetched and artificial [37].

Most conformational optimization methods in docking program can only deal with a single objective, such as binding energy, shape complementarity, or chemical complementarity. However, real-world problems normally involve multiple objectives (possibly conflicting ones) or optimization criteria, which should be satisfied simultaneously, and suitable solutions to the overall problem cannot be found by using individual optimization algorithms with single objectives[38]. For example, an optimization solution for the binding affinity (energy) between a ligand and a receptor is usually not the optimization solution for other criteria (e.g. shape or chemical complementarity, etc.). Similar problems in combinatorial library design and structural superposition of three-dimensional molecules have been reported[39,40]. Thus, there is a need for an optimization algorithm compromising several objectives, which may result in more reasonable and robust binding modes between ligands and receptors. In fact, it is a problem of multi-objective optimization (MO)[41], which tends to find a set of alternative good compromises, generally known as *pareto-optimal *solutions. These solutions are optimal because no other solutions in the search space superior to them when all objectives are considered. Then, the 'optimum' is chosen by the design which fits better in a certain application.[42] There are more than twenty mathematical multi-objective optimization techniques[43,44]. However, due to their inherent parallelism, evolutionary algorithms (EAs) and genetic algorithms (GAs) are still the top priority in terms of finding multiple *pareto-optimal *solutions for multi-objective optimization problems[45].

In this paper, two sets of MO methods, denoted MOSFOM (Multi-Objective Scoring Function Optimization Methodology), were adopted for the binding conformation search of a small molecule within the binding site of a protein using two scoring functions as the objectives. Different from consensus score, MOSFOM does not re-score or re-rank the candidates from the primary virtual screening with one or several other scoring functions, but scores all the molecules in a chemical database with two or more scoring functions simultaneously during the binding pose optimization. Testing results indicate that MOSFOM, which is able to enhance hit rates and greatly reduces the false-positive rate, is more robust and reasonable than the consensus strategy as an alternate tool for large-scale library virtual screening. Here, MOSFOM emphasizes a new strategy to obtain the most reasonable binding conformation[46] and increase hit rates with several scoring functions rather than to accurately predict the binding free energy or the combination of several scoring functions. Consequently, MOSFOM can be used in the prioritization of ligands in high-throughput virtual screening.

## Methods

### Preparation of target proteins

Thrombin, the estrogen receptor alpha, and cyclooxygenase-2 (COX-2) have been used as target proteins for testing the newly developed docking algorithms in this study. The coordinates of the X-ray crystal structures of these three proteins were retrieved from the Protein Data Bank (PDB)[47], including thrombin in complex with Mqpa at 2.2 Å resolution (PDB entry 1ETR)[48], estrogen receptor(ER) in complex with 4-hydroxytamoxifen at 1.90 Å resolution (PDB entry 3ERT)[49], and cyclooxygenase-2(COX-2) in complex with Sc-558 at 3.0 Å resolution (PDB entry 6COX)[50]. All water molecules were removed from the protein structures. After extraction of bound ligands, all hydrogen atoms and the Kollman all-atom charges were assigned to the proteins using the BIOPOLYMER module of Sybyl v6.8 (Tripos Associates, Inc. St. Louis, MO). Finally, for each protein target, the binding site was defined as the residues around the bound ligand within 6.5 Å. Gasteiger and Marsili charges[51] were assigned to the extracted ligand of each protein.

### Preparation of compounds libraries

For all three targets (thrombin, ER and COX-2), the active compounds were selected from the MDDR (MDL ISIS/HOST software, MDL Information Systems, Inc.). Active compounds with molecular weights between 200 and 600 were selected as drug-like compounds, and those containing water molecules and ions were eliminated. Table 1 shows the number of active compounds for each target. Another 10,000 randomly 'varied' compounds with molecular weights between 200 and 600 were selected as drug-concerned decoys from the Available Chemical Directory (ACD) after eliminating chemical reagents and inorganic compounds by means of CORINA. All compounds of different test libraries were stored as SDF format file using the MDL ISIS_Base program (MDL ISIS/HOST software, MDL Information Systems, Inc.), and their three-dimensional coordinates were converted using a script written in the Sybyl programming language (SPL), and Gasteiger-Marsili atomic charges were assigned to each molecule. The final coordinates of each molecule were then stored in multi-mol2 files. Protonation states were correctly given for all the compounds.

**Table 1:** Number of the active compounds for each target used in this study

Target	MW Cutoff	No. Actives	No. Selected	Range RB (Mean)	Theoretical Maximum of Enrich Factor
Thrombin	200–600	847	646	4–29 (12.4)	16.47
ER	200–600	134	105	3–17 (8.5)	96.23
COX-2	200–600	698	695	0–17 (4.0)	15.39
Random database	200–600		10000	0–35 (4.5)	

### Scoring functions

The energy score and contact score of DOCK [52-54] were used in this work. Energy score based on the AMBER force field[55] was composed of steric and electrostatic terms. *ε*(r) = 4r was used for the coefficient of the dielectric for the Coulomb potential, and Lennard-Jones 6–12 was used for Van der Waals (VDW) potential. Contact score is a summation of the heavy atom contacts between the ligand and the receptor, which provides a simple assessment of shape complementarity; if two atoms approach close enough to bump into one another, then the interaction can be penalized by a certain amount. In this study, a 4.5-Å contact distance cutoff was used, with 50 penalized for each clash.

### Calculation of the enrichment factor

The enrichment factor is a key parameter to evaluate the quality of the docking and scoring compared to a random selection[33,56]. The enrichment factor (EF) is defined as

(1) $$\text{EF(subset size)}=\frac{{\text{Hits}}_{\text{s}}}{{\text{N}}_{\text{s}}}/\frac{{\text{Hits}}_{\text{t}}}{{\text{N}}_{\text{t}}}$$

where Hits_s _is the number of active compounds in the sampled subset, Hits_t _is the total number of active compounds in the database and N is the number of compounds. In general, the enrichment factor is against random screening; it is evident that the maximum enrichment is determined by the total number of active compounds and the total number of molecules in the database. For instance, there are 695 active compounds among the total 10695(10000+695) molecules in the database for the COX-2 case, i.e. the achievable maximum is 10695/695 ≈ 15. If 5% of active compounds were found among the top 1% of the database, then the enrichment factor would be fivefold over random (EF = 5) at the 1% of the database.

There are three criteria to evaluate the effectiveness of a docking program as indicated by Wei *et al*. [56]: the value, the location of the vertex, and the percentage of active compounds retrieved, which represent the ability to find active compound of the docking program and scoring function.

### *ε*-MOEA Method

A steady-state MOEA based on the *ε*-dominance concept[57] is a pragmatic and fast multi-objective evolutionary algorithm for finding well-spread *pareto-optimal *solutions. An EA population *P*(t) and an archival population *E*(t) (t is the iteration counter) were used as two co-evolving populations in *ε*-MOEA. After initialization, two solutions from *P*(t) and *E*(t) were chosen for mating. Then, each of these offspring solutions was compared with the archive and the EA population for possible inclusion. For the case of *j *objectives, the search space and objective space were divided into hyper-boxes (a number of grids, each having the size *ε*_*j *_in the *j*th objective) to ensure that a hyper-box could be occupied by only one solution through comparing with an identification array of the archive, which guarantees the diversity of the archive. The total number of *pareto-optimal *solutions, i.e. the final size of suitable solutions, can be controlled approximately by adjusting the *ε*_*j *_value (see refs [58] and [57] for more details).

**Figure 1:**
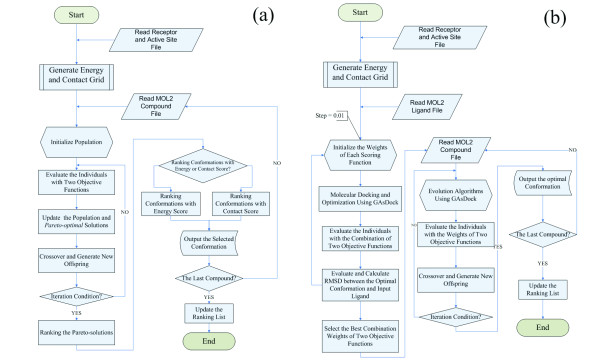
**The flowchart of Multi-Objective Scoring Function Optimization Methodology**. (a) ε-MOEA method for virtual screening. (b) EFMOGA method for virtual screening.

Two scoring functions, the energy score and the contact score of DOCK, were considered in practical virtual screening. Traditional optimization methods, such as the simplex method used in DOCK, are unsuitable for multi-objective optimization. For the impartiality of the comparison, GAsDock, which also uses a stochastic algorithm based on modified multi-population genetic algorithm[25], was adopted for single scoring function optimization. The contact score also performed reasonably well in this study, which coincides with other studies [31,59,60]. Energy score and contact score were considered as the two objectives in *ε*-MOEA (Figure 1a), that is, a set of *pareto-optimal *solutions, which were satisfied simultaneously with energy score and shape complementarity, were obtained consequently (see additional file 1: Docking example and proof of method).

The multi-objective optimization model of *ε*-MOEA consists of a set of *n *parameters (design variables), a set of *l *objective functions, and a set of *m *constraints. Objective functions and constraints are functions of the decision variables. It can be formulated mathematically as follows:

(2) $$\begin{array}{ll} min & y=f\text{(}x\text{)}=\text{(}f_{\text{1}}(x),f_{2}(x),...,f_{l}(x))\\ s.t. & g(x)=(g_{1}(x),g_{2}(x),...,g_{m}(x))\leq 0\\ where & x=(x_{1},x_{2},...,x_{n})\in X\\ & y=(y_{1},y_{2},...,y_{l})\in Y \end{array}$$

and ***x ***is the design vector ***x ***= {*T*_*x*_, *T*_*y*_, *T*_*z*_, *R*_*x*_, *R*_*y*_, *R*_*z*_, *T*_*b*1_, ⋯ *T*_*bn*_}^T^, in which (*T*_*x*_, *T*_*y*_, *T*_*z*_) and (*R*_*x*_, *R*_*y*_, *R*_*z*_) are respectively the state variables of translation and rotation of the entire ligand for the orientation search; and *T*_*b1*_, ⋯, *T*_*bn *_are the torsion angles of the *n *rotatable bonds of the ligand for the conformation search. Accordingly, the constraints for the design variables (***g****(****x****)s*) can be represented as

(3) $$\{\begin{array}{c} \underline{X}\leq T_{x}\leq \bar{X}\\ \underline{\;Y}\leq T_{y}\leq \bar{Y}\\ \underline{\;Z}\leq T_{z}\leq \bar{Z}\\ -\pi \leq R_{x},R_{y},R_{z},T_{b1},\cdots ,T_{bn}\leq \pi \end{array}$$

***y ***is the objective vector, which consists of energy score ***f***_1_(***x***) and contact score ***f***_2_(***x***), respectively. Of course, more scoring function can be used in this method, but no more scoring function source can be accessed. Here the scoring functions of DOCK were just used to deal with this problem. ***X ***is denoted as the decision space, and ***Y ***is called the objective space.

### Selection of the optimum and ranking in *ε*-MOEA case

As stated above, a set of *pareto-optimal *solutions were obtained by using *ε*-MOEA, which simultaneously satisfied energy score and shape complementarity. There are two ways to select an optimal solution from the set of *pareto-optimal solutions*: MOEA_Nrg or 'energy score ≻ contact score' with the lowest energy conformation and acceptable shape complementarity; and MOEA_Cnt or 'contact score ≻ energy score' with the best shape complementarity conformation and acceptable energy score. The results of MOEA_Nrg and MOEA_Cnt were all compared with their corresponding individual scoring functions, respectively (see Results)

### EFMOGA

Briefly, a new fast flexible docking program (GAsDock) [25,61] was developed using a multi-population genetic algorithm based on information entropy[62,63]. In comparison with other docking methods, information entropy was employed in the genetic algorithm of GAsDock and the size of the narrowed space was used as the convergence criterion, ensuring that GAsDock can converge rapidly and steadily. A detailed description of the algorithm has been presented elsewhere[25].

In this paper, EFMOGA-based GAsDock was applied to solve the above-mentioned multiple scoring function problem. According to GAsDock, the optimization problem (Eq.2) can be transformed into the following evaluation function model

(4) $$\begin{array}{ll} min & h(F)=\frac{1}{s}\ln\sum_{i=1}^{l}\exp(s\lambda_{i}f_{i}(x))\\ s.t. & g(x)=(g_{1}(x),g_{2}(x),...,g_{m}(x))\leq 0\\ where & x=(x_{1},x_{2},...,x_{n})\in X\\ & y=(y_{1},y_{2},...,y_{l})\in Y \end{array}$$

where *λ*_*i *_is the weight of each objective. (*λ*_*i *_≥ 0 and $\sum_{i=1}^{l}\lambda_{i}=1(i=1,2,...,l)$). By varying the weights, a set of noninferior solutions was generated. *s *is a positive real variable, when *s *→ ∞, the minimization problem converges to the maximal *f*_*i*_(x), and the minimum is the *pareto *solution (see additional file 1: Docking example and proof of method)

A knowledge-based method was adopted to obtain appropriate weights for virtual screening, i.e. a set of conformations of the ligand, which are obtained through adjusting the weights, is compared with the ligand in the X-ray crystal structure, and the weights with minimum RMSD are selected. These are considered to best reflect the test values for the current target system, i.e. the most reasonable solution. During this process, useful information can be acquired simultaneously such as the adaptability of different scoring functions to the current system, that is, which scoring function contributes more to the current system, and which scoring function is more sensitive.

As a result, various weights are obtained for different test systems in virtual screening (Figure 1b). For virtual screening of a database, each system only adopts one set of weights, which makes the comparison more equitable. Different from *ε*-MOEA, EFMOGA yields one solution corresponding to the weight in the *pareto-optimal *solutions rather than a set of *pareto-optimal *solutions. Certainly, compared with single objective optimization, the solution obtained here may be worse than any single objective solution, but more reasonable than any extremum obtained by a single objective method because it has considered multiple-objective functions. During the preparation of this manuscript, Grosdidier et al. have developed a new docking software, EADock, based on a multi-objective optimization algorithm, and the results indicate that EADock can accurately predict binding modes for ligand-protein complexes with two fitness functions[64].

## Results

As mentioned above, our study is not aimed at calculating absolute values for the free energy of binding and for the affinity, but focuses on the ranking of acceptable conformations for the multiple solution space. Because of the different preferences in the selection of *pareto-optimal *solutions with *ε*-MOEA, the results of three different approaches will be compared with individual scoring functions, namely one with preferred energy score for *ε*-MOEA (MOEA_Nrg), the other with preferred contact score for *ε*-MOEA (MOEA_Cnt), and the third for EFMOGA.

### Docking to the buried lipophilic site of COX-2

**Figure 2:**
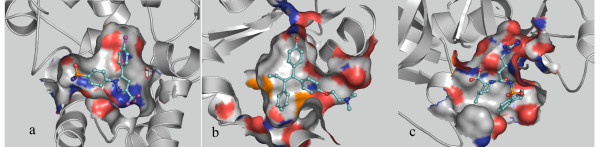
**The characteristics of three binding sites**. COX-2 with Sc-558. (b) ER with 4-Hydroxytamoxifen. (c) Thrombin with Mqpa.

**Figure 3:**
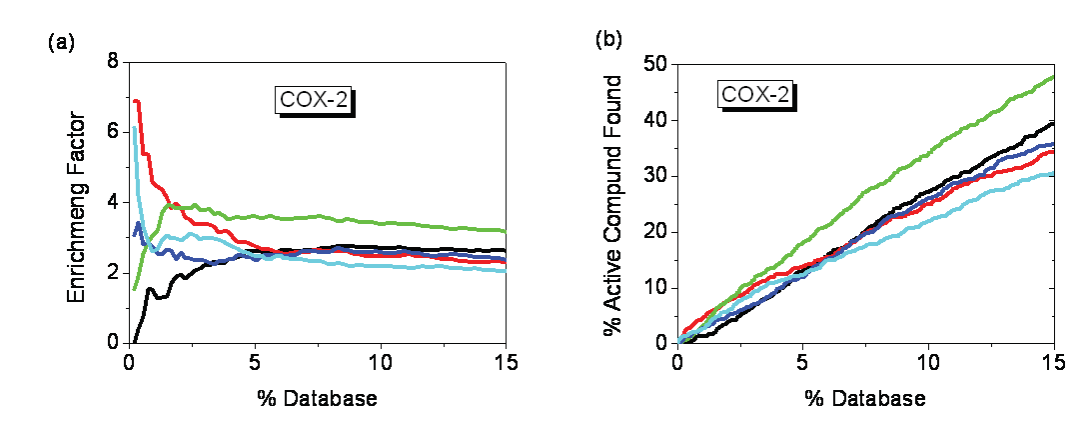
**Enrichment of 695 active compounds of COX-2 in docking screens**. (a) The enrichment factor of docking the database by MOFOM and individual scoring function. (b) The percentage of the active compounds found by MOFOM and individual scoring function. The plot shows the results using five optimization methods: Energy score (black), Contact score (red), EFMOGA (cyan), MOEA_Nrg (green), and MOEA_Cnt (blue), respectively.

The COX-2 ligand binding site is a completely buried, narrow, confined and predominately lipophilic cavity (Figure 2a)[50,65], which does not accommodate many orientations or conformations of a ligand. Consequently, shape complementarity was expected to be very important. MOEA_Nrg efficiently eliminates conformations that could not satisfy shape complementarity although they displayed good energy scores. The maximum enrichment was 2.8-fold over random, which was reached at the top 8.8% of the database when using a single energy score for optimization, but the maximum enrichment was 3.9-fold over random, and moved up to the top 2.6% of the database when using MOEA_Nrg (Figure 3a). MOEA_Cnt also got a good enrichment (EF = 3.4485) at the top 0.4% of the database, but it was worse than single contact score with EF = 6.9 at the top 0.2%. There are several possible explanations; first, the conformation with highest contact score cannot satisfy energy score, that is, the conformation that has the best contact score but a bad energy score not among the *pareto-optimal *solutions will be eliminated; second, the scoring function is imprecise which is the common disadvantage for all the scoring functions. Interestingly, although contact score maintained higher enrichment among the top 2% of the database, the same enrichment as that of MOEA_Nrg was obtained at the top 2% of the database, and from this point, the enrichment peak of MOEA_Nrg became broader. EFMOGA has a similar curve as that of MOEA_Cnt, and a maximum of 6.1-fold over random was reached at the top 0.2% of the database.

**Figure 4:**
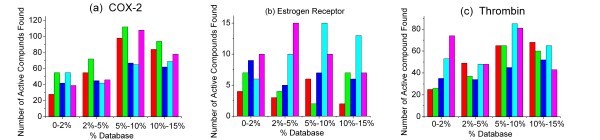
**Histogram of active compounds found with five MOFSOM and single scoring function for assaying between the 2%, 5%, 10% and 15% of the ranked database against each system**. (a) COX-2 system, (b) Estrogen receptor system, (c) Thrombin system with five optimization methods: Energy score (red), Contact score (cyan), EFMOGA (blue), MOEA_Nrg (green), and MOEA_Cnt (pink), respectively.

In the real application, only a small number (< 2%) was our interesting section within a large source library. MOEA_Nrg exhibited a good performance in this case (Figure 3b, Figure 4a), MOEA_Nrg maintained the best performance among all the methods, and retrieved 7.8% of the COX-2 active compounds within the top 2% of the total library (Figure 3b, Figure 4a). EFMOGA outperformed single energy score among the top 5% of the library, and retrieved 6% of active compounds among the top 2% of the database, although it performed worse than single contact score. Whereas, MOEA_Cnt represents a relatively weak ability than single contact score before the top 5% of the database, but greatly increases thereafter.

### Docking to the relatively large hydrophobic site of Estrogen Receptor

As an example of a well-understood target with therapeutic relevance whose crystallographic structural data were publicly available, estrogen receptor was chosen. Like COX-2, the binding site of estrogen receptor is a relatively larger, fully-enclosed lipophilic cavity (Figure 2b), which is little opened to solvent, there are acceptor groups at either end that can form hydrogen bonds with ligands, but it is predominantly hydrophobic on the whole[49,65].

**Figure 5:**
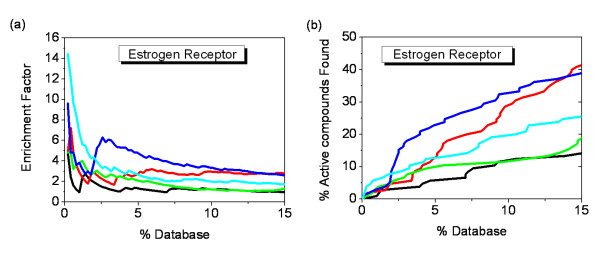
**Enrichment of 105 active compounds of ER in docking screens**. (a) The enrichment factor of docking the database by MOFOM and individual scoring function. (b) The percentage of active compounds found by MOFOM and individual scoring function. The plot shows the results using five optimization methods: Energy score (black), Contact score (red), EFMOGA (cyan), MOEA_Nrg (green), and MOEA_Cnt (blue), respectively.

For this case, EFMOGA performed well among the top 5% of the database, especially before the top 2%, and reached the maximum enrichment (EF = 14.4) at the top 0.2% of the database (Figure 5a). Single energy score exhibited the poorest performance in all methods, the maximum enrichment is less than fivefold over random at the top 0.2% of the database, although MOEA_Nrg reached the same enrichment, MOEA_Nrg outperformed single energy score after that. Different from COX-2, MOEA_Cnt indicated a very good performance than single contact score for this case, it is more reasonable that we preferred contact score from *pareto*-*optimal *solutions to select the better shape satisfaction with slight difference in energy score. Using single contact score, the maximum enrichment (EF = 7.2) was reached at the top 0.4% of the database, but the maximum enrichment rose to 9.6, and moved up to the top 0.2% of the database when using MOEA_Cnt.

Similar to COX-2, approximately 5.7% of active compounds were retrieved using MOEA_Nrg at the top 2% of the database (Figure 5b, Figure 4b), which was about twofold over that of single energy score. Relative to single contact score, MOEA_Cnt retrieved 9.5% of the active compounds at the top 2% of the database, but only less than 5% of the active compounds were retrieved using single contact score. It is encouraging that EFMOGA represents better performance than energy score and contact score at the top 2% of the database, with 8% of active compounds retrieved.

### Docking to the intermediate polarity site of Thrombin

In contrast to COX-2 and estrogen receptor, the binding site of thrombin is an intermediate polarity site[65], with a large hydrophobic pocket (a smaller proximal and a larger distal pocket) which is a more exposed binding site to solvent[48], in addition, there is a S1 specificity pocket, which is a narrow and restricted pocket comprising the carboxylate group of Asp189 at the bottom, most thrombin inhibitors form hydrogen bonds with Asp189 and also to Gly216 (Figure 2c). Due to many polar groups in the binding site, it is difficult to distinguish active compounds for DOCK energy score which is most reliable for the apolar active site, unlike those scoring functions with extra consideration of hydrogen bonding interactions such as PMF or FlexX score[18,34,65-67].

**Figure 6:**
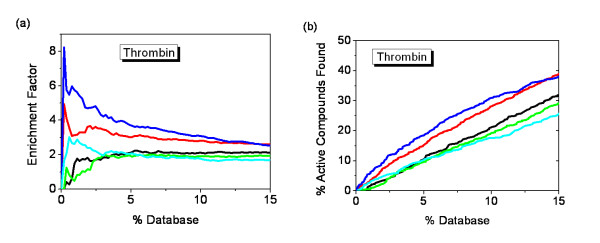
**Enrichment of 646 active compounds of thrombin in docking screens**. (a) The enrichment factor of docking the database by MOFOM and individual scoring function. (b) The percentage of active compounds found by MOFOM and individual scoring function. The plot shows the results using five optimization methods: Energy score (black), Contact score (red), EFMOGA (cyan), MOEA_Nrg (green), and MOEA_Cnt (blue), respectively.

As expected, although the maximum enrichment was 2 at the top 6.4% of the database for MOEA_Nrg, and the maximum enrichment was 2.2 at the top 5.5% of the database when using single energy score, there is a slight increase in quantity using MOEA_Nrg against single energy score at the top 2% of the database (Figure 6a). MOEA_Nrg slightly underperformed than single energy score after the top 2% of the database (Figure 6b). Like ER case, MOEA_Cnt performed the best in all the methods, the maximum enrichment (EF = 8.3) was reached at the top 0.2% of the database, approximately twofold over contact score, with a maximum value of 4.9 at the top 0.2% of the database (Figure 6a), at the same time, 9.4% of the active compounds were retrieved with MOEA_Cnt at the top 2% of the database (Figure 4c). Same as other two systems, EFMOGA continues a good performance at the top 2% of the database, with a 3.0 enrichment reached at the top 0.6% of the database, and retrieved approximately 5% of the active compounds at the top 2% of the database, which is less than that of contact score, but better than that of energy score, EFMOGA and MOEA_Nrg performed not so well compared with single score function after the top 5% of the database.

## Discussion

### Performance of MOSFOM and Characteristics of binding site and preferential selection of scoring function

Simple as it is, contact score performed unexpectedly well for all the three systems in our study. Especially at the top 5%, contact score performed better than energy score, possibly arising from molecular diversity of the randomly selected ACD database, which distributes well not only in heavy atoms but also molecular torsions. Contact score can rapidly seek out those whose geometry shape can satisfy the binding site (containing shape and volume), which is especially obvious to those with completely buried and narrow cavity. However, energy score presented a weak ability to distinguish the active compounds from the decoys with shape satisfaction.

It is encouraging that compared with individual score, every method of MOSFOM presented an increase not only in enrichment but also hit rates of the active compounds retrieved in some ways. EFMOGA with different weights in different systems obtained better results than single score at the top 5% (especially at the top 2%) of the database for all three cases in this study (see Results), but gradually faded afterwards. It can be easily understood that those active compounds with high affinity and good shape complementarity were first retrieved, and then those emphasizing particularly on the larger weight in different test system were obtained, consecutively. Therefore, if not knowing which scoring function will work better for the test system in advance, more enrichment can be obtained with EFMOGA in the most interesting section (generally 2%) of the database. It should be noted that EFMOGA, with different weights for different test systems through experimental protein-ligand complex, is more reasonable and different from the simple linear combination (LC)[68] of several scoring functions or ScreenScore through a combination of scoring terms[69]. EFMOGA seems more pertinent and advantageous, and will be a tendency for scoring and ranking to consider different scoring functions focusing on diverse aspects for different systems.

Different from EFMOGA, *ε*-MOEA selected the preferential solution from a *pareto-optimal *solutions family (parts of the *pareto *frontier) through multi-objective optimization using multiple scoring functions (see Methods). With different preferences, MOEA_Nrg and MOEA_Cnt all outperformed their corresponding individual scoring function, i.e., MOEA_Nrg corresponding to energy score and MOEA_Cnt corresponding to contact score. A higher enrichment and hit rates of the active compounds were obtained using MOEA_Nrg than using individual energy score in COX-2 and ER system, but there is an inverse phenomenon for thrombin case. There are several strong hydrogen bond interactions between the ligand and the residues in S1 pocket for thrombin system. If we select the one with lowest energy score from the docked conformations with acceptable shape complementarity, more compounds will have good shape complementarity conformations but are only slightly different in energy score, since the energy score does not pay more additional attention to the H-bond interaction. In this case, it will be more difficult to distinguish the active compounds. On the contrary, MOEA_Cnt performed well for the thrombin and ER case, where an open cavity exists, accommodating enough orientations or conformations of a ligand, therefore, it seems more reasonable that MOEA_Cnt selected the best shape complementarity conformation with differences in energy score. So, if one does know which scoring function will work better for the test system in advance, more enrichment and better performance can be obtained using *ε*-MOEA through relative preference.

### Limitations of MOSFOM, further improvements and prospect in bioinformatics and drug design

More scoring functions, which pay special attention to hydrogen bonding interaction, or chemical complementarity, solvent effect, should be involved in multi-objective optimization, however, there are only energy score and contact score available for us. Although MOSFOM performed better than individual score, there will be more choices for MOFSOM in specific test system if more interactions were considered. We can determine different weights of scoring functions because EFMOGA can be determined to deal with different cases and maybe prefer others (e.g. chemical complementarity or hydrogen bonds) for *ε*-MOEA if we know which scoring function will work well for specific test system in advance. Therefore, to develop scoring functions with focus on hydrogen bond, icon or solvent effect and to utilize multi-objective optimization to acquire more reasonable conformations is one of our future research interest.

At the same time, ignorance of the impact of protein flexibility is another limitation for MOSFOM, small changes of the receptor flexibility result in larger variety of binding affinities[70], and docking to a single receptor conformation will significantly reduce the chance of finding the correct pose. Considering protein flexibility to acquire correct protein-ligand binding conformation is crucial for medicinal chemists to find out the structure-activity relationship. We will fulfil this work in the future. Also, computational time of multi-objective optimization is another issue to be solved. There are numerous multi-objective optimization methods, however, they are not suitable for large-scale virtual screening because of the huge time consumption, in this paper, *ε*-MOEA, which is a steady-state MOEA based on the *ε*-dominance concept, fulfilled virtual screening quickly within about one minute for one molecule, and EFMOGA, which is a multi-population MOGA based on information entropy, accomplished one docking within 40 seconds using one CPU on SGI origin3800 hardware. However, more improvements are needed to find a set of well-distributed solutions close to the true *pareto *frontier pragmatically and efficiently.

As stated above, multi-objective optimization approach has been adopted in the library design program MoSELECT[39,71-73] and quantitative structure activity relationships program MoQSAR[74]. We believe that MOEA or MOGA also can be applied to biological sequence alignment, protein fold recognition, conformational generation and ADME/TOX (absorption, distribution, metabolism, excretion and toxicity) with more and more factors taken into account in bioinformatics and drug design.

## Conclusion

Development of a fast and accurate scoring function in virtual screening is still a hot and pending issue in current research, different scoring functions focus on different aspects of ligand binding, and no single scoring can satisfy all the systems well, therefore, consensus score was put forward.[35,36] With several other scoring functions, consensus score re-assessed those conformations optimized using a primary scoring function, but it is not really robust from the viewpoint of optimization. All of these give rise to another heuristic thinking for us, is it possible and more rational to find a most reasonable conformation in the process of optimization using two or more scoring functions in virtual screening? Since present scoring functions can not satisfy all the cases, multi-objective optimization method can be a good compromise.

In this study, we present two kinds of multi-objective optimization method, called MOSFOM, in virtual screening which simultaneously considers energy score and contact score. A set of *pareto*-*optimal *solutions were obtained which can simultaneously satisfy energy and shape complementarity using *ε*-MOEA method, then through two different preferences, the binding conformations of *pareto-optimal *solutions with lowest energy scoring or best shape complementarity were ranked among all selected conformations. However, EFMOGA acquires different weights of each scoring function for different systems based on experimental X-ray complex structure, that is, for different system, EFMOGA regards the varying weight as the degree of contribution of individual scoring function, which generally focuses on one aspect for a special type of binding sites, this means the scoring function with bigger weight is more suitable for the binding site. We used 10000 random compounds as the decoys, active compounds selected from MDDR database were randomly merged into the decoys, respectively. To ensure justness of comparison, GAsDock, based on an improved genetic algorithm, was used as a benchmark for single-objective optimization. Results show that MOSFOM can enhance the enrichment and greatly increase the hit rates compared with individual score (see Results). For three different kinds of binding sites, MOSFOM represents excellent ability of distinction of active compounds with energy and shape complementarity. EFMOGA specially performed well at the top 2% of the database, MOEA_Nrg and MOEA_Cnt performed better than individual scoring function if a proper type of binding site was selected.

Multi-objective optimization method was successfully applied in virtual screening with two different scoring functions, which can gain reasonable binding pose, and can be furthermore ranked with those potentially compromised conformations of each compound which abandon those conformations that can not satisfy overall objective functions. By analyzing the characteristics of binding site in advance, the most effective multi-objective optimization method is adopted, which is meaningful for all current scoring functions since they can not suit all cases. More specific scoring functions and protein flexibility will be taken into account in our future work.

## Authors' contributions

HL, HZ and JL contributed to the development and validation of the method, MZ, LK, and XL contributed to the analysis and data interpretation, XW and HJ conceived the idea of the method, and provided guidance for its development and revised the subsequent drafts of this manuscript. All authors read and approved the final manuscript.

## Supplementary Material

Additional file 1 **Docking example and method proof.** A molecular docking example for reproduction of Thymidine Kinase (TK) complex, and the mathematical proof for evaluation function multi-objective optimization genetic algorithm (EFMOGA). Click here for file
