# Supplementary material for: An effective docking strategy for virtual screening based on multi-objective optimization algorithm
Source: BMC Bioinformatics. 2009 Feb 11;10:58. doi: 10.1186/1471-2105-10-58 (PMC2753843; doi:10.1186/1471-2105-10-58)
Supplement: Additional file 1 — Docking example and method proof. A molecular docking example for reproduction of Thymidine Kinase (TK) complex, and the mathematical proof for evaluation function multi-objective optimization genetic algorithm (EFMOGA). [file 1471-2105-10-58-S1.pdf]

Appendix

Reproduction of Thymidine Kinase (TK) Complex

Thymidine kinase (PDB entry: 1kim) in complex with dT (deoxythymidine) was chosen for evaluation of reproduction, protein and ligand were prepared as the Materials. Using ε-MOEA, a set of *pareto-optimal* solutions were obtained (Figure S1a), all of which simultaneously satisfied energy score and shape complementarity. For MOEA_Nrg and MOEA_cnt, the conformations with the lowest energy and best shape complementarity were selected from the *pareto-optimal* solutions, RMSD (Root Mean Square Deviation) compared with the conformation of X-ray complex was 0.21 Å and 0.55 Å, respectively (Figure S2b). Similarly, the set of *pareto-optimal* solutions were obtained by EFMOGA with variational weights for the two objective scoring functions (Figure S1b). The conformation was selected with the smallest RMSD value (0.22 Å) shown in Figure S2c, the weights of each scoring function were decided at the same time, which if needed will be used for the virtual screening for the TK system.

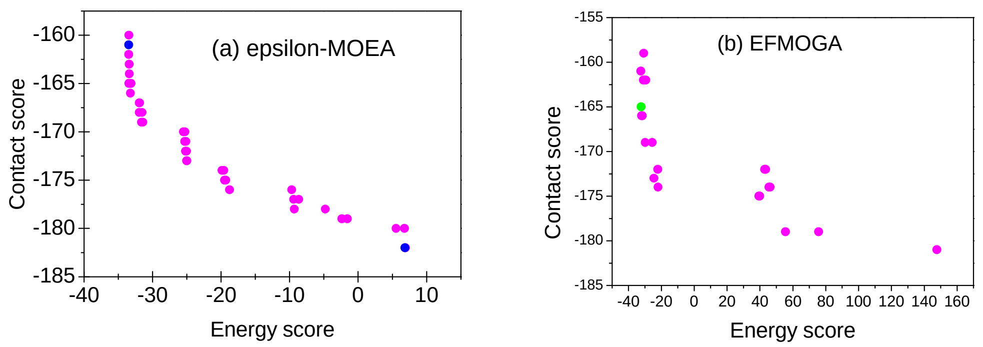

Figure S1. Distribution of *pareto-optimal* solutions with the MOSFOM. (a) ε-MOEA, the blue points are the selected solution with MOEA_Nrg and MOEA_Cnt,

respectively. (b) EFMOGA, the green point is the selected solution with smallest RMSD value (0.22 Å) in the *pareto-optimal* solutions.

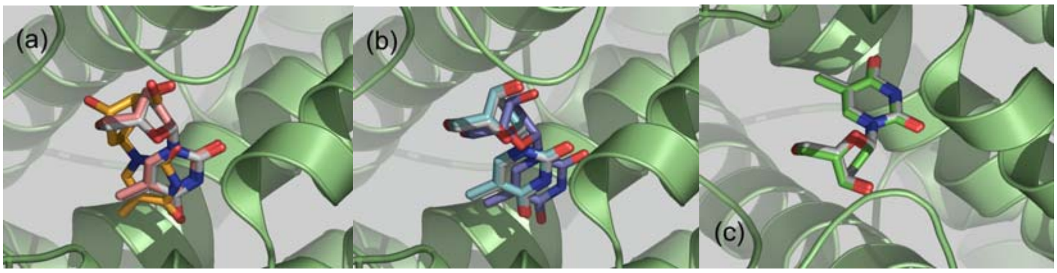

Figure S2. Docking of dT into the 1KIM binding pocket. (a) The docked poses by GAsDock with single scoring function. RMSD values compared with X-ray pose are 0.23 Å and 2.64 Å to energy score and contact score, respectively. (b) The docked pose of MOSFOM by ε-MOEA method. RMSD values are 0.21 Å and 0.55 Å to MOEA_Nrg and MOEA_Cnt. (3) The docked pose of MOSFOM by EFMOGA method with a RMSD value of 0.22 Å. The following color coding was used: X-ray pose, white carbon atoms; energy score pose, incarnadine carbon atoms; contact score pose, yellow carbon atoms; MOEA_Nrg pose, blue carbon atoms; MOEA_Cnt pose, cyan carbon atoms; EFMOGA pose, green carbon atoms.

Mathematical proof for evalucation function multiobjective optimization genetic algorithm

Theorem: if $s \to \infty$, then the optimization problems

$$Minimize\{Maximize\ f_i(\mathbf{x})\},\ i = 1,\cdots,q \tag{A1}$$

and

$$Minimize\ \ h(F) = \frac{1}{s}\ln\sum_{i=1}^{q}\exp(s\lambda_i f_i(\mathbf{x})) \tag{A2}$$

are equivalent.

Prof. The s norm of the q -dimensional vector

$$H_F = \left[e^{f_1(\mathbf{x})}, e^{f_{21}(\mathbf{x})}, \cdots, e^{f_q(\mathbf{x})}\right]^{\mathrm{T}} \tag{A3}$$

is given by

$$N_t(H_F) = \left[\sum_{i=1}^{q} e^{tf_i(\mathbf{x})} \right]^{1/s} \tag{A4}$$

The uniform norm, also called the maximum norm, is defined by

$$N_\infty(H_F) = \lim_{s \to \infty} N_t(H_f) \tag{A5}$$

Since $e^{f_i(x)} > 0$ by Jensen's inequality, the norm is a strictly monotone decreasing function of its order, i.e.

$$N_t < N_r \quad \text{for } r < t \tag{A6}$$

Taking logarithms on both sides of equation (A6) and substituting from equations (A4) and (A5) gives

$$N_\infty(H_F) = Max\left[e^{f_i(\mathbf{x})}\right] < N_r(H_F) \tag{A7}$$

$$\lim \frac{1}{s}\left\{ \ln\left[\sum_{i=1}^{q} \exp(sf_i(\mathbf{x}) \right] \right\} = Max\left[f_i(\mathbf{x})\right] \tag{A8}$$

Minimizing both sides of equation (A8) with respect to design variables ***x***,

$$\lim_{s \to \infty} Min\, h(F) = Min\{Max\left[f_i(\mathbf{x})\right]\} \tag{A9}$$

Note that λ is constant, so

$$\lim_{s \to \infty} \left\{ Min \frac{1}{s} \ln \sum_{i=1}^{q} \exp(s\lambda_i f_i(\mathbf{x})) \right\} = Min\{Max\left[f_i(\mathbf{x})\right]\} \tag{A10}$$

and the proof is completed.
